# Recommendations for the use of clinical outcome assessments in rare disease drug development

**DOI:** 10.1016/j.eclinm.2026.104073

**Published:** 2026-07-20

**Authors:** Olalekan Lee Aiyegbusi, Noleen K. McCorry, Melanie J. Calvert, John Devin Peipert, Samantha Cruz Rivera, Robert Muni-Lofra, Solomon Alexis, Fez Awan, Tom Bailey, Lucinda Billingham, Rosaline Callaghan, Christine Collins, Brooke M. Currie, Mel Dixon, Melanie Duddridge, James Ennis, Linda Fred, Sarah Greenwell, Christian Griffiths, Asha Hareendran, Onyekachukwu A. Illoh, Kerry Leeson-Beevers, Krishna Letchemanan, Steven P. Lister, Christopher McCabe, Molly McFatrich, Amy Jayne McKnight, Fiona McLaughlin, Ramona Moldovan, Lindsey T. Murray, Daniel J. O'Connor, Hafiz Oko-osi, Louise Oni, Kate M. Pritchard, Khadija Rerhou Rantell, Volker Straub, Timothy G. Barrett, LifeArc Accelerating Rare Disease Trials (ARDT) centreaf, David Jones, Michael Clarke, Hayley Comins, Simon Gates, Amber R. Hart, Victoria Hedley, Blánaid Hicks, Martin R. Higgs, Matthew Hosken, Ameeta Retzer, Sarah Scullion, Laura A. Wyatt, Steven J. Blackburn, Catherine Turner

**Affiliations:** a Centre for Patient Reported Outcomes Research, Department of Applied Health Sciences, College of Medicine and Health, University of Birmingham, Birmingham, UK; b Birmingham Health Partners Centre for Regulatory Science and Innovation, University of Birmingham, Birmingham, UK; c National Institute for Health and Care Research (NIHR) Birmingham Biomedical Research Centre, University of Birmingham, Birmingham, UK; d National Institute for Health and Care Research (NIHR) Applied Research Collaboration (ARC) West Midlands, University of Birmingham, Birmingham, UK; e NIHR Blood and Transplant Research Unit (BTRU) in Precision Transplant and Cellular Therapeutics, University of Birmingham, Birmingham, UK; f LifeArc Centre for Acceleration of Rare Disease Trials, University of Birmingham, Birmingham, UK; g LifeArc Centre for Acceleration of Rare Disease Trials, Queen's University Belfast, Belfast, UK; h Centre for Public Health, Queen's University Belfast, Belfast, UK; i The John Walton Muscular Dystrophy Research Centre, Translational and Clinical Research Institute, Newcastle University, Newcastle, UK; j Newcastle Hospitals NHS Foundation Trust, Newcastle, UK; k University of East London, London, UK; l LifeArc Centre for the Acceleration of Rare Disease Trials, Newcastle University, Newcastle, UK; m Alexion, AstraZeneca Rare Disease, London, UK; n GlaxoSmithKline, Collegeville, PA, USA; o National Institute for Health and Care Excellence (NICE), Manchester, UK; p University of Bedfordshire, Luton, UK; q US Food and Drugs Administration, Silver Spring, MD, USA; r Alström Syndrome UK, Devon, UK; s Union Chimique Belge (UCB) Pharma SA, Slough, UK; t Queen's Business School, Queen's University Belfast, Belfast, UK; u Takeda Pharmaceutical, Chapel Hill, NC, USA; v University of Manchester, Manchester, UK; w Critical Path Institute, Tucson, AZ, USA; x Association of the British Pharmaceutical Industry (ABPI), London, UK; y BioMarin Pharmaceutical Inc., San Rafael, CA, USA; z UCL Centre for Kidney and Bladder Health, London, UK; aa University of Liverpool, Liverpool, UK; ab Department of Paediatric Nephrology, Great Ormond Street Hospital, London, UK; ac Medicines and Healthcare products Regulatory Agency (MHRA), London, UK; ad Faculty of Medical Sciences, Newcastle University, Newcastle, UK; ae Centre for Rare Disease Studies, University of Birmingham, Birmingham, UK

**Keywords:** Clinical outcome assessment, Patient-reported outcome, PRO, Rare diseases, Clinical trials, Patient-focused drug development

## Abstract

There are over 10,000 rare diseases collectively affecting an estimated 250–450 million people globally. While these diseases are rare individually, their cumulative impact on patients, families, healthcare systems, and society is substantial. The incorporation of clinical outcome assessments (COAs) in clinical trials can facilitate patient-focused drug development and treatment evaluation by generating meaningful evidence on how patients feel and function. This work was conducted in three phases: a targeted literature review (searched Aug 2025; updated Feb 2026), a multistakeholder workshop (online, Sept 2025) and, finally, an online survey to ratify final recommendations (responses by March 3, 2026). Of 43 individuals invited, 35 (81%) attended the virtual workshop: 11 researchers (including clinical trialists); 12 patients/caregivers; seven industry experts; four individuals from regulatory agencies and one HTA expert. All were based in the UK or USA. Across three sessions, the workshop explored stakeholder perspectives on considerations and appropriate methodological approaches to COA assessment for rare disease drug development to facilitate the generation of recommendations for future use. A threshold of at least 70% votes was chosen, a priori, for inclusion in the final set of recommendations. Here, we describe the potential benefits of COAs, summarise the key challenges, and provide recommendations to facilitate their effective and consistent integration in drug development for rare diseases.

## Introduction

A rare disease is a condition that affects a small percentage of the population, typically defined as fewer than 1 in 2000 people in the UK and European Union or affecting fewer than 200,000 people in the United States.^1^^,^^2^ It is estimated that there are over 10,000 rare diseases affecting an estimated 250–450 million people worldwide.^3^^,^^4^ Approximately 80% of rare diseases have a genetic cause, with about 70% of these presenting in childhood.^3^^,^^4^ Fewer than 5% of rare diseases have approved pharmacological therapies.^3^ While rare diseases are individually uncommon, they have substantial impacts on the lives of those affected.^4^, ^5^, ^6^, ^7^, ^8^, ^9^, ^10^, ^11^, ^12^, ^13^, ^14^, ^15^ The economic costs are also substantial. A recent European survey reported that the aggregated annual economic cost of 43 rare diseases affecting approximately 1.9 million people across the nine European countries surveyed (France, Germany, Italy, the Netherlands, Poland, Romania, Spain, Sweden, and the United Kingdom) was estimated as €249 billion in 2023.^16^ The estimated annual aggregate cost for the US was approximately $960 billion in 2019.^17^

In recognition of the personal, economic, and societal impacts of rare diseases, as well as the sustained campaigning by patients, their families/caregivers, and patient organisations, governments, regulatory agencies, and health technology assessment (HTA) bodies have implemented a range of initiatives to encourage the development of therapies for rare diseases.^1^^,^^18^, ^19^, ^20^, ^21^, ^22^ There is also an increased drive for global collaboration between governmental institutions, academia, patient organisations, and industry to accelerate patient-focused drug development for rare diseases.

Patient-focused drug development (PFDD) is a systematic approach to ensure that patients' experiences, perspectives, needs, and priorities are captured and meaningfully incorporated into therapy development and evaluation.^23^, ^24^, ^25^, ^26^ The US Food and Drugs Administration (FDA) has developed four PFDD guidance documents to facilitate the collection and submission of patient experience data (PED) and other relevant information from patients and caregivers for drug development and regulatory decision making.^23^, ^24^, ^25^, ^26^ PED collected using scientifically rigorous methods including clinical outcome assessments (COAs), qualitative research, surveys and patient preference studies generate evidence for the evaluation of new treatments.^23^^,^^27^, ^28^, ^29^, ^30^ Four types of COAs, namely patient-reported outcomes (PROs), clinician-reported outcomes (ClinROs), observer-reported outcomes (ObsROs), and performance outcomes (PerfOs) are used to evaluate a patient's health status by measuring how they feel, function, or survive (see Table 1 for definitions).^25^^,^^26^^,^^31^, ^32^, ^33^

**Table 1:** Definitions and explanations of acronyms.

Acronym	Full name	Definition/Explanations
COA	Clinical outcome assessment	A tool or method used to measure and reflect changes in how a patient feels, functions, serving as a key metric in evaluating the effectiveness of medical interventions in clinical trials and improving patient care. There are four main types of COAs: patient-reported outcomes (PROs), clinician-reported outcomes (ClinROs), observer-reported outcomes (ObsROs), and performance-based outcome measures (PerfOs). See the descriptions of these below.^25^
ClinRO	Clinician-reported outcome	This is a type of clinical outcome assessment and include clinically observable signs, behaviours, and clinical manifestations of the disease. For example, a clinician listens to wheezing during a lung exam. Patients don't typically listen to their own lungs.^25^
PRO	Patient reported outcome	These are reports provided by patients about their own health, quality of life, or functional status associated with health care or treatment. ePROs are electronically collected PROs.^25^
PROMs	Patient-Reported Outcome Measures	These are standardised questionnaires patients complete to provide direct feedback on their health status, symptoms, well-being, and quality of life without interpretation or proxy reporting by anyone else. For example, a questionnaire could measure a patient's rating of the severity of pain.^25^
ObsRO	Observer-reported outcomes	A clinical outcome assessment based on a report of observable signs, events, or behaviours related to a patient's health condition by someone other than that patient or a health professional. Generally, ObsROs are reported by a parent, caregiver, or someone who observes the patient in daily life and are particularly useful for patients who cannot report for themselves (e.g., infants or individuals who are cognitively impaired).^25^
PerfO	Performance-based outcome	A performance-based outcome is a measurable result that reflects a person's ability to perform a specific task or movement, such as a patient completing a timed physical activity.^25^
PPIE	Patient and public involvement and engagement	• The **‘involvement’** component of Patient and Public Involvement and Engagement (PPIE) refers to activities and research carried out ‘with’ or ‘by’ members of the public or patients, rather than ‘to’, ‘about’ or ‘for’ them. Patients and members of the public are actively involved in the development, running and management of research projects or activities.^31^^,^^32^ • The **‘engagement’** element of PPIE focuses on the dissemination of information and outcomes from research to patients and the public, so that they are informed of developments while providing them the opportunity to share their insights and input.^31^^,^^32^ • **Coproduction** is “an approach in which researchers, practitioners and the public work together, sharing power and responsibility from the start to the end of the project, including the generation of knowledge.” Patients, members of the public and other stakeholders are equal partners in research with joint ownership of key decisions during the project.^33^

However, COAs are under-utilised and sub-optimally implemented in drug development for rare diseases.^34^ A recent review of FDA orphan labels for new molecular entities and biologic licence applications with FDA orphan designations found that PRO was included in only 13.5% of labels between 2018 and 2024.^35^ This is a modest increase of 5.2% from the level reported for the 2002 to 2017 period.^35^ Although limited data on the use of ObsROs in rare disease drug development exists, there are indications that these are utilised less frequently than PROs.^20^ These findings highlight the need for improvement in the utilisation of COAs in rare disease drug development for patient benefit.^36^^,^^37^

Here, we describe the potential benefits of COAs in rare disease drug development, summarise the key challenges, and provide recommendations to facilitate the utilisation of COAs in rare disease drug development. The recommendations were developed based on the findings from a literature review and a multi-stakeholder workshop. Table 2 lists the workshop attendees while a list of useful resources and guidance documents are provided in Table 3.

**Table 2:** Workshop attendance list.

First name	Middle name or initial	Surname	Affiliations	Stakeholder group	Location
A. Participants					
Sarah		Greenwell	LifeArc Accelerating Rare Disease Trials (ARDT)	Patients & Caregivers	UK
Linda		Fred	LifeArc ARDT	Patients & Caregivers	UK
Fez		Awan	LifeArc ARDT	Patients & Caregivers	UK
James		Ennis	LifeArc ARDT	Patients & Caregivers	UK
Kate	M	Pritchard	LifeArc ARDT	Patients & Caregivers	UK
Mel		Dixon	LifeArc ARDT	Patients & Caregivers	UK
Melanie		Duddridge	LifeArc ARDT	Patients & Caregivers	UK
Fiona		McLaughlin	LifeArc ARDT	Patients & Caregivers	UK
Rosaline		Callaghan	LifeArc ARDT	Patients & Caregivers	UK
Christine		Collins	LifeArc ARDT	Patients & Caregivers	UK
Kerry		Leeson-Beevers	LifeArc ARDT	Patients & Caregivers	UK
Solomon		Alexis	University of East London, London, UK	Patients & Caregivers	UK
Noleen	K	McCorry	LifeArc ARDT; Centre for Public Health, Queen's University Belfast, UK	Researcher/clinical trialist	UK
David		Jones	Newcastle University; LifeArc ARDT	Researcher/clinical trialist	UK
Timothy	G	Barrett	University of Birmingham; LifeArc ARDT	Researcher/clinical trialist	UK
Melanie	Jane	Calvert	Centre for Patient Reported Outcomes Research, University of Birmingham, LifeArc ARDT	Researcher/clinical trialist	UK
Louise		Oni	University College London Centre for Kidney and Bladder Health, London; University of Liverpool; LifeArc	Researcher/clinical trialist	UK
Hareendran		Asha	University of Bedfordshire	Researcher/clinical trialist	UK
Lucinda		Billingham	University of Birmingham, LifeArc ARDT	Researcher/clinical trialist	UK
Volker		Straub	Newcastle University; LifeArc ARDT	Researcher/clinical trialist	UK
Ramona		Moldovan	University of Manchester, Manchester, UK	Researcher/clinical trialist	UK
Christopher		McCabe	Centre for Public Health, and Queen's Business School, Queen's University Belfast, UK; LifeArc ARDT	Researcher/clinical trialist	UK
Amy Jayne		McKnight	Centre for Public Health, Queen's University Belfast, UK; LifeArc ARDT	Researcher/clinical trialist	UK
Lindsey	T	Murray	Critical Path Institute, Tucson, AZ, USA	Regulatory/HTA/Non-profit expert	USA
Onyekachukwu	A	Illoh	United States Food and Drug Administration (FDA)	Regulatory/HTA/Non-profit expert	USA
Khadija		Rerhou Rantell	Medicines and Healthcare products Regulatory Agency (MHRA)	Regulatory/HTA/Non-profit expert	UK
Christian		Griffiths	National Institute for Health and Care Excellence (NICE)	Regulatory/HTA/Non-profit expert	UK
Daniel	J	O'Connor	Association of the British Pharmaceutical Industry (ABPI)	Industry expert	UK
Tom		Bailey	Medical Affairs, Alexion, AstraZeneca Rare Disease	Industry expert	UK
Molly		McFatrich	Takeda Pharmaceutical, Chapel Hill, North Carolina, USA	Industry expert	USA
Steven	P	Lister	Union Chimique Belge (UCB) Pharma SA	Industry expert	UK
Brooke	M	Currie	GlaxoSmithKline (GSK)	Industry expert	USA
Hafiz		Oko-osi	BioMarin Pharmaceutical Inc.	Industry expert	USA
Krishna		Letchemanan	Country Operations, Alexion, AstraZeneca Rare Disease	Industry expert	UK
B. Observers					
Amber	R	Hart	Newcastle University; LifeArc ARDT		UK
Matthew		Hosken	Newcastle University; LifeArc ARDT		UK
Sarah		Scullion	Queen's University Belfast, UK; LifeArc ARDT		UK
Laura	A	Wyatt	University of Birmingham; LifeArc ARDT		UK
C. Moderator					
Olalekan	Lee	Aiyegbusi	Centre for Patient Reported Outcomes Research, University of Birmingham, LifeArc ARDT	Researcher/clinical trialist	UK

∗Patients & Caregivers—included members of the Lived Experience Advisory Panel (LEAP) for the LifeArc ARDT, patient advocates, and other individuals with lived experience of rare diseases from the personal networks of the study team.

∗∗A workshop participant (Regulatory/HTA/Non-profit expert), who is not a co-author, requested to remain anonymous.

**Table 3:** Guidance to support the use of Clinical Outcome Assessments (COAs) in rare disease drug development.

Trial design	Calvert, M. et al. Guidelines for Inclusion of Patient-Reported Outcomes in Clinical Trial Protocols: The SPIRIT-PRO Extension. JAMA. 2018 Feb 6; 319 (5):483–494. https://doi.org/10.1001/jama.2017.21903.
	Benjamin, K. et al. Patient-Reported Outcome and Observer-Reported Outcome Assessment in Rare Disease Clinical Trials: An ISPOR COA Emerging Good Practices Task Force Report. Value Health. 2017 Jul–Aug; 20 (7):838–855. https://doi.org/10.1016/j.jval.2017.05.015.
	Nestler-Parr, S. et al. Challenges in Research and Health Technology Assessment of Rare Disease Technologies: Report of the ISPOR Rare Disease Special Interest Group. Value Health. 2018 May; 21 (5):493–500. https://doi.org/10.1016/j.jval.2018.03.004.
	Matza, L.S. et al. Paediatric patient-reported outcome instruments for research to support medical product labelling: report of the ISPOR PRO good research practices for the assessment of children and adolescents task force. Value Health. 2013 Jun; 16 (4):461-79. https://doi.org/10.1016/j.jval.2013.04.004.
	Calvert, M. et al. SPIRIT-PRO Extension explanation and elaboration: guidelines for inclusion of patient-reported outcomes in protocols of clinical trials. BMJ Open. 2021 Jun 30; 11 (6):e045105. https://doi.org/10.1136/bmjopen-2020-045105.
	Yap, C. et al. Advancing patient-centric care: integrating patient reported outcomes for tolerability assessment in early phase clinical trials—insights from an expert virtual roundtable. *eClinicalMedicine* 76, 102838 (2024).
	Amdal, C. D. et al. SISAQOL-IMI consensus-based guidelines to design, analyse, interpret, and present patient-reported outcomes in cancer clinical trials. The Lancet Oncology 26 (12): e683-e693. DOI: htps://doi.org/10.1016/S1470-2045(25)00520-0
	Day, S. et al. Recommendations for the design of small population clinical trials. *Orphanet Journal of Rare Diseases* 13, 195 (2018).
	Carona, C. et al. Applying a developmental approach to quality of life assessment in children and adolescents with psychological disorders: challenges and guidelines. Expert Rev Pharmacoecon Outcomes Res. 2015 Feb; 15 (1):47–70. https://doi.org/10.1586/14737167.2015.972377.
	Chen, J. et al. Challenges and Possible Strategies to Address Them in Rare Disease Drug Development: A Statistical Perspective. *Clin Pharmacol Ther* 118, 62–73 (2025).
	Banerjee, A.K. et al. Patient-Reported Outcome Measures in Safety Event Reporting: PROSPER Consortium guidance. Drug Saf. 2013 Dec; 36 (12):1129-49. https://doi.org/10.1007/s40264-013-0113-z.
	Powers, J.H. et al. Clinician-Reported Outcome Assessments of Treatment Benefit: Report of the ISPOR Clinical Outcome Assessment Emerging Good Practices Task Force. Value Health. 2017 Jan; 20 (1):2–14. https://doi.org/10.1016/j.jval.2016.11.005.
	Rothman, M. et al. Use of existing patient-reported outcome (PRO) instruments and their modification: the ISPOR Good Research Practices for Evaluating and Documenting Content Validity for the Use of Existing Instruments and Their Modification PRO Task Force Report. Value Health. 2009 Nov–Dec; 12 (8):1075-83. https://doi.org/10.1111/j.1524-4733.2009.00603.x.
	Edgar, C.J. et al. Recommendations on the Selection, Development, and Modification of Performance Outcome Assessments: A Good Practices Report of an ISPOR Task Force. Value in health: the journal of the International Society for Pharmacoeconomics and Outcomes Research 26, 959–967 (2023).
Repository of patient-reported outcome (PRO) guidance for clinical trials	Proteus Consortium: https://theproteusconsortium.org
Electronic PRO (ePRO) systems	Zbrozek, A. et al. Validation of electronic systems to collect patient-reported outcome (PRO) data-recommendations for clinical trial teams: report of the ISPOR ePRO systems validation good research practices task force. Value Health. 2013 Jun; 16 (4):480-9. https://doi.org/10.1016/j.jval.2013.04.002.
	Aiyegbusi, O.L. Key methodological considerations for usability testing of electronic patient-reported outcome (ePRO) systems. Qual Life Res. 2020 Feb; 29 (2):325–333. https://doi.org/10.1007/s11136-019-02329-z.
	Hudgens, S. et al. Best Practice Recommendations for Electronic Patient-Reported Outcome Dataset Structure and Standardisation to Support Drug Development. Value Health. 2023 Aug; 26 (8):1242–1248. https://doi.org/10.1016/j.jval.2023.02.011.
	Ly, J.J. et al. Training on the Use of Technology to Collect Patient-Reported Outcome Data Electronically in Clinical Trials: Best Practice Recommendations from the ePRO Consortium. Ther Innov Regul Sci. 2019 Jul; 53 (4):431–440. https://doi.org/10.1177/2168479018796206.
	O'Donohoe, P. et al. Considerations for Requiring Subjects to Provide a Response to Electronic Patient-Reported Outcome Instruments. Ther Innov Regul Sci. 2015 Nov; 49 (6):792–796. https://doi.org/10.1177/2168479015609647.
Artificial intelligence	Cruz Rivera, S. et al. Guidelines for clinical trial protocols for interventions involving artificial intelligence: the SPIRIT-AI extension. *Nature Medicine* 26, 1351–1363 (2020).
	Fleurence, R.L. et al. Generative Artificial Intelligence for Health Technology Assessment: Opportunities, Challenges, and Policy Considerations: An ISPOR Working Group Report. *Value in Health* 28, 175–183 (2025).
Addressing respondent burden	Aiyegbusi, O.L. et al. Recommendations to address respondent burden associated with patient-reported outcome assessment. *Nature Medicine* (2024).
	Aiyegbusi, O.L. et al. Key considerations to reduce or address respondent burden in patient-reported outcome (PRO) data collection. *Nature Communications* 13, 6026 (2022).
Translation of Clinical Outcome Assessments (COA)	McKown, S. et al. Good practices for the translation, cultural adaptation, and linguistic validation of clinician-reported outcome, observer-reported outcome, and performance outcome measures. *Journal of Patient-Reported Outcomes* 4, 89 (2020).
COA interpretation	Nicod, E. et al. Improving Interpretation of Evidence Relating to Quality of Life in Health Technology Assessments of Rare Disease Treatments. *The Patient - Patient-Centered Outcomes Research* 16, 7–17 (2023).
Regulatory guidance	FDA. Patient-Focused Drug Development: Collecting Comprehensive and Representative Input. Guidance for Industry, Food and Drug Administration Staff, and Other Stakeholders. (U.S. Department of Health and Human Services Food and Drug Administration, Silver Spring, MD, 2020).
	FDA. Patient-Focused Drug Development: Methods to Identify What Is Important to Patients. Guidance for Industry, Food and Drug Administration Staff, and Other Stakeholders. (U.S. Department of Health and Human Services Food and Drug Administration, Silver Spring, MD, 2022).
	FDA. Patient-Focused Drug Development: Selecting, Developing, or Modifying Fit-for Purpose Clinical Outcome Assessments. Guidance for Industry, Food and Drug Administration Staff, and Other Stakeholders. (US Food and Drug Administration, Silver Spring, MD, 2025).
	FDA. Patient-Focused Drug Development: Incorporating Clinical Outcome Assessments Into Endpoints For Regulatory Decision-Making. Guidance for Industry, Food and Drug Administration Staff, and Other Stakeholders. (U.S. Department of Health and Human Services Food and Drug Administration, Silver Spring, MD, 2023).
	EMA. Qualification of novel methodologies for drug development: guidance to applicants. (2014).
COA qualification European Medicines Agency (EMA)	Silva, M. et al. Patient-reported, observer-reported and performance outcomes in qualification procedures at the European Medicines Agency 2013–2018. *British Journal of Clinical Pharmacology* 90, 299–312 (2024).
Reporting COA data	Calvert, M. et al. Reporting of patient-reported outcomes in randomised trials: the CONSORT PRO extension. JAMA. 2013 Feb 27; 309 (8):814-22. https://doi.org/10.1001/jama.2013.879.
	Snyder, C. et al. Making a picture worth a thousand numbers: recommendations for graphically displaying patient-reported outcomes data. Qual Life Res. 2019 Feb; 28 (2):345–356. https://doi.org/10.1007/s11136-018-2020-3.
Equity, Diversity and Inclusion (EDI) and Patient and public involvement and engagement (PPIE)	Retzer, A. et al. A toolkit for capturing a representative and equitable sample in health research. *Nature Medicine* 29, 3259–3267 (2023).
	Calvert, M.J. et al. Patient reported outcome assessment must be inclusive and equitable. *Nat Med* 28, 1120–1124 (2022). https://doi.org/10.1038/s41591-022-01781-8
	Aiyegbusi, O.L. et al. Recommendations to promote equity, diversity and inclusion in decentralised clinical trials. *Nature Medicine* (2024).
	Petkovic, J. et al. Health Equity Considerations for Developing and Reporting Patient-reported Outcomes in Clinical Trials: A Report from the OMERACT Equity Special Interest Group. J Rheumatol. 2017 Nov; 44 (11):1727–1733. https://doi.org/10.3899/jrheum.160975.
	Aiyegbusi, O.L. et al. Considerations for patient and public involvement and engagement in health research. Nature Medicine (2023).
Ethical considerations	Cruz Rivera, S. et al. Ethical Considerations for the Inclusion of Patient-Reported Outcomes in Clinical Research: The PRO Ethics Guidelines. JAMA. 2022 May 17; 327 (19):1910–1919. https://doi.org/10.1001/jama.2022.6421.
	Arbuckle, L. et al. Montreal Accord on Patient-Reported Outcomes (PROs) use series - Paper 9: anonymization and ethics considerations for capturing and sharing patient reported outcomes. J Clin Epidemiol. 2017 Sep; 89:168–172. https://doi.org/10.1016/j.jclinepi.2017.04.016.

## Methods

### Study approach and ethics

This work was conducted in three phases starting with a targeted literature review, followed by a multistakeholder workshop, and finally a survey completed by the co-authors to ratify the final recommendations.

Original qualitative data in the form of workshop discussions was collected for this study. Plans for the workshop were reviewed and approved by the STEM ethics committee at the University of Birmingham, UK (Reference number: ERN_4863-Aug2025). Informed consent was obtained from the workshop participants who completed and signed individual consent forms. A verbal consent was also obtained at the start of the workshop.

### Targeted literature review

#### Justification

A targeted literature review was done rather than a systematic review for three main reasons. First, our primary aim was to obtain an overview of the topic and ensure that sufficient relevant and current evidence was considered to support the workshop and the subsequent recommendations and not to conduct an exhaustive search to capture all existing literature that may be relevant. Second, the decision to conduct a targeted literature review was pragmatic as it is less resource intensive (time and personnel) and suitable for finding specific information for the rapid evidence assessments which we required for our work. Finally, based on previous experience, we knew that a significant proportion of relevant documents will be grey literature such as regulatory guidance and will not be found by searching multiple databases.^38^, ^39^, ^40^, ^41^ Therefore, the literature search was conducted by OLA in two steps—a PubMed database search followed by a pearl growing strategy (also known as “snowballing”) using Google Scholar.^38^, ^39^, ^40^

#### Search strategy and selection criteria

PubMed was searched by OLA on Aug 26, 2025 to identify relevant articles. There were no restrictions on study design or language of publication. However, the search was restricted to articles published within the last 10 years to capture the most recent evidence on clinical outcome assessment in rare disease clinical trials. The Supplementary File provides details of the search strategy, the entries retrieved, the eligibility criteria, the selection process, data analysis and synthesis. The PubMed search was updated on Feb 27, 2026 by SCR.

#### Pearl growing strategy

OLA utilised the pearl growing strategy to identify additional relevant articles including grey literature.^38^^,^^39^ As the 2017 ISPOR report was highly relevant to our work, it was used as a ‘pearl’ for the backward and forward citation mining on Google Scholar. This search was conducted on the Aug 28, 2025.

### Multistakeholder workshop

#### Justification

To drive progress in COA in rare disease drug development, greater evidence of the effectiveness of several strategies or approaches is urgently required. This can only be determined if they are all implemented and robustly evaluated. Therefore, it is essential that each recommendation we provide is sufficiently considered for implementation by stakeholders without prejudging their relative importance particularly as every rare disease context is unique. This meant that the Delphi method and nominal group technique were not suitable as they typically involve voting based on opinions of the relative importance of suggestions or recommendations and only those ranked the highest are carried forward.

#### Workshop details

The virtual workshop was hosted by the University of Birmingham, UK, on behalf of LifeArc Centre for Acceleration of Rare Disease Trials (LifeArc ARDT) on Microsoft Teams on Sept 5, 2025. LifeArc ARDT is a consortium of Queen's University Belfast, Newcastle University, and University of Birmingham. Members of the core study team (OLA, NKM, DJ, TGB, JDP, LAW, SS) created a spreadsheet of the names, expertise, and contact details of potential workshop participants who belonged to at least one of the four stakeholder groups. These potential participants were identified through their personal networks and recommendations from other researchers, industry and regulatory experts. The stakeholder groups were: (1) members of the Lived Experience Advisory Panel (LEAP) for the LifeArc ARDT; other individuals with lived experience of rare diseases from the personal networks of the study team; (2) researchers/clinical trialists; (3) industry experts, and (4) regulatory and HTA experts.

Workshop participants were selected from this spreadsheet based on the requirement that they have lived experiences of a rare disease either as patients or as family members/caregivers of patients with rare diseases and/or professional expertise and experience of COA and rare disease research. Although not formally ascertained, demographic characteristics such as gender and ethnicity and stakeholder group representation were also considered during the selection process. The LEAP members and other patients who attended the workshop reflected the breadth of rare diseases including metabolic, renal, respiratory, haematological, musculoskeletal, autoimmune/rare immunodeficiencies, and neurodegenerative rare diseases.

The study team sent workshop invitations and information on the study aim and objectives, and consent forms to 43 individuals via email. Invitation acceptances were proactively monitored to ensure the diversity of the workshop attendees in terms of gender, ethnicity, and stakeholder group taking into consideration the potential intersectionality of participants’ characteristics.^42^

The workshop explored stakeholder perspectives on considerations and appropriate methodological approaches to COA assessment for rare disease drug development to facilitate the generation of recommendations for future use. Pre-meeting materials, detailing the workshop objectives and the draft literature review were sent to participants beforehand.

The workshop discussions were organised as three sessions all on the same day, involving all the workshop participants. Session 1 focused on what outcomes to measure and how this should be done. Important considerations for COA for rare disease clinical trials were discussed during Session 2 while Session 3 explored future developments in outcome measurement in clinical trials. The key issues and considerations identified in the literature review were discussed during the relevant session. The moderator (OLA) led the discussions, starting with a key question for each session and used prompts when required although spontaneous, unguided responses were encouraged. The moderator deliberately refrained from expressing his own preferences or views during the discussions to avoid introducing bias. While participants had the option of using the Teams chat function to contribute, the moderator called on individuals when necessary to expand verbally on their chat comments.

Further details of the workshop discussions and how they map to the recommendations can be found in the Supplementary File.

### Generation and review of draft recommendations

Sequential steps taken to develop the draft recommendations were:

1. OLA and MJC independently took notes of the key issues and suggestions/recommendations raised at the workshop in addition to the audio recording.
2. At the end of the workshop, they held a debrief meeting where they discussed their notes.
3. During this debrief meeting, OLA created an initial outline of the issues and potential recommendations.
4. OLA then listened to the audio recording and the chat transcript to ensure that all the important points and suggestion were covered by the outline recommendations. He also noted the points made by participants that were unclear and wrote separately to the relevant participants for clarifications.
5. OLA mapped the literature review findings to the draft recommendations and where needed, searched PubMed using free text for additional evidence or guidance to support and further develop/refine the draft recommendations.
6. BMC and HO separately documented, summarised, and emailed OLA their reflections and notes they made of the key points discussed during workshop. OLA then checked that these as well as the pre-workshop comments from DJO were captured in the draft recommendations.
7. OLA conducted a final check listening to the audio recording.
8. The initial report of eleven recommendations generated based on findings from the literature review and workshop discussions were sent for review by co-authors (workshop participants and other individuals) who provided feedback and suggested two additional recommendations.
9. The initial report of recommendations was revised by OLA, and two new recommendations were added based on the feedback received from co-authors.

### Final consultation

**Fig. 1:**
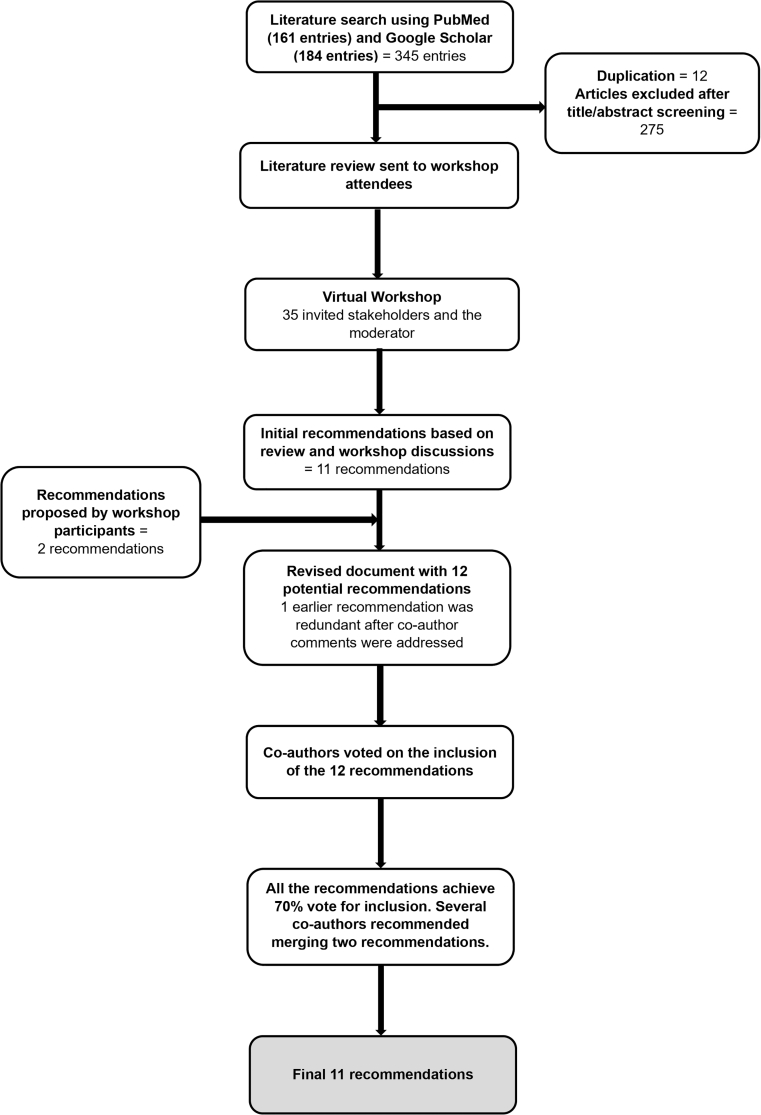
Flow diagram.

The core study team (OLA, NKM, DJ, TGB, JDP, LAW, SS) decided that even though co-author feedback was generally supportive of the recommendations, it was important to formally evidence this through a vote. Following the revision of the initial report of recommendations, an online survey with ‘yes’ and ‘no’ options for each recommendation (including the two additional ones suggested by the workshop participants) was created by SCR using Smart Survey. The survey questionnaire is available within the Supplementary File. Workshop participants had between Feb 9, and March 3, 2026 to complete the survey. A threshold of at least 70% votes was chosen a priori for the inclusion in the final set of recommendations. The survey link and the revised report of recommendations were circulated to co-authors for voting, final comments and approval for publication. The voting data was analysed by SCR and the report of recommendations finalised by OLA for submission. Fig. 1 illustrates the process of generating the recommendations. The Supplementary File provides further details of the voting.

### Study-level management of potential conflicts of interest

As a preventative measure, potential participants were informed when they were invited that the goal of the study was to produce broad recommendations applicable across the rare diseases without focussing on any particular disease or pharmaceutical products. Furthermore, the pre-meeting information provided to the participants explicitly stated that: *“Participants are free to illustrate their points using any rare disease they are familiar with. However, the purpose of this workshop is to generate and capture information and insights applicable across rare diseases.”* This point was reiterated at the start of the workshop. The a priori decision if any participant inadvertently mentioned or recommended a specific treatment or drug for any specific rare disease during the workshop or while reviewing the report of recommendations was to anonymise this information in the final report. However, this was not necessary as none of the participants made such comments.

### Role of the funding source

The funder, LifeArc, was not involved in study design, data collection, data analyses, data interpretation, generation of recommendations, or the writing of the manuscript.

## Results

### Topline summary

The targeted literature review, on Aug 26, 2025, identified 161 articles on PubMed; of these, 20 were relevant (including the 2017 ISPOR COA Task Force Report on Rare Disease Clinical Trials.^43^) The updated PubMed search, on Feb 27, 2026, yielded 28 additional articles; two of these were relevant and were incorporated into the review. The pearl growing strategy utilised the 2017 ISPOR report. Backward and forward citation mining, on Aug 28, 2025, identified 184 citing articles. Of these, 62 were relevant to this study. Of the 43 individuals invited to participate in the virtual workshop, 35 individuals (81%) attended. These were 11 researchers (including clinical trialists); 12 patients/caregivers; seven industry experts; four individuals from regulatory agencies and one HTA expert. Also in attendance were four observers (non-participating) and the moderator (OLA). Table 2 provides details of workshop attendees. Full details of the workshop discussions and how they map to the recommendations, outlined below, can be found in the Supplementary File. For the final consultation, the survey was completed by 28 (78%) of the 36 co-authors invited, which included 10 patients/patient advocates/caregivers, eight researchers/clinical trialists, six industry experts, three regulatory and HTA experts, and one self-identified COA expert. All of the 12 recommendations (10 initial and two additional recommendations suggested by co-authors) achieved the a priori threshold of at least 70% votes for inclusion. Several co-authors recommended merging two recommendations which resulted in 11 final recommendations. Full details of the voting results can be found in the Supplementary File.

### Potential benefits of COAs in rare disease drug development

COA provide evidence of efficacy and tolerability of new therapies as primary and or secondary endpoints in clinical trials.^44^, ^45^, ^46^, ^47^, ^48^, ^49^, ^50^, ^51^, ^52^ For instance, the PRUCISION ObsRO measure was used to assess the primary endpoint of proportion of positive pruritus assessments (PPAs) over 24 weeks in the randomised, placebo-controlled, phase 3 trial of odevixibat for the treatment of progressive familial intrahepatic cholestasis (PFIC).^52^ In addition, PRUCISION ObsRO and PRO measures were used to assess secondary endpoints. PFIC is a group of inherited paediatric liver diseases that impact bile secretion leading to pruritus which can considerably reduce health-related quality of life (HRQOL).^52^ The ObsRO and PRO data showed that treatment with odevixibat led to statistically significant improvements compared with placebo over the treatment period.^52^ This corroborated the results for the second primary endpoint of serum bile acid response.

Furthermore, the interim efficacy data obtained using the composite (ClinRO and PerfO) measure, Mullen Scales of Early Learning (MSEL), showed significant improvements in developmental outcomes in the Phase1/2 trial of an oligodendrocyte-targeted adeno-associated virus gene therapy for Canavan disease.^48^ The inclusion of COA data in New Drug Applications for rare diseases may provide valuable evidence to inform decision-making by regulatory and HTA agencies.^37^^,^^53^ An example is the approval of Brineura (cerliponase alfa) for the treatment of neuronal ceroid lipofuscinosis type 2 (CLN2) disease. The drug was approved by the EMA and FDA based on the primary outcome data collected using the CLN2 Clinical Rating Scale, a performance outcome (PerfO) measure, in the pivotal trial.^49^ Another example is the FDA's assessment, regulatory decision, and labelling of acoramidis for the treatment of adult patients with cardiomyopathy due to wild-type or variant transthyretin-mediated amyloidosis. The FDA considered the secondary endpoint data assessed using the Kansas City Cardiomyopathy Questionnaire-Overall Summary score (KCCQ-OS), (a PRO), which showed that treatment with acoramidis led to a significant change in health status from baseline to month 30 in a Phase III trial.^54^

COA data are considered relevant outcomes in the Population, Intervention, Comparator, Outcomes (PICO) evaluation framework of HTA agencies such as the Scottish Medicines Consortium (SMC), the Zorginstituut Nederland, the National Institute for Health and Care Excellence (NICE), England, and Joint Clinical Assessment (JCA), allowing for a deeper understanding of disease burden and treatment impact, and a broader assessment of the effectiveness and safety/tolerability of health technologies beyond objective clinical endpoints thereby facilitating decisions regarding healthcare policies^37^^,^^44^^,^^53^^,^^55^ COA data can also be an important component of post-marketing evidence requirements for rare disease therapies approved via the accelerated approval pathway.^49^ Real world COA data collection offers the opportunity to overcome the limitations of small sample sizes that challenge rare disease clinical trials through access to more respondents and more frequent measurement.

### Challenges with incorporating COAs

The challenges associated with conducting clinical trials for rare diseases and incorporating COAs in this context have been well described.^4^^,^^5^^,^^34^^,^^43^^,^^56^, ^57^, ^58^, ^59^, ^60^, ^61^ Although many of these challenges are not exclusive to rare diseases, they are amplified by the characteristics of the individual patient populations (i.e., typically small, heterogenous, geographically dispersed). Due to the relatively small numbers of patients living with each rare disease, there is often limited awareness, knowledge and experience of supporting individuals with rare conditions.^4^^,^^61^, ^62^, ^63^ The small size of patient populations may result in uncertain aggregate trial results.^64^ In rare disease populations, emotional well-being may improve simply due to the sense of hope and validation from participating in a trial (research participation effects).^65^ Disentangling true treatment effects from research participation effects, particularly for emotional, mental, or psychological outcomes can be challenging especially as randomised placebo-controlled studies are often prohibited in rare disease drug development, requiring reliance on data from single arm trials.^65^

The limited or lack of knowledge about the natural histories of many rare diseases, disease heterogeneity and variability make it challenging to select outcomes that can be evaluated as meaningful clinical trial endpoints that are generalisable across the entire spectrum of a rare disease.^43^^,^^58^^,^^66^^,^^67^ This can impact on the ability of regulators and payers to confidently model the effect of therapy for all patients with the clinical indication leading to approval for some sub-groups in regulatory and/or HTA considerations.

Furthermore, patients with rare diseases may be young children or have cognitive impairments linked to their disease, making self-reporting of PROs difficult (or impossible), and requiring the use of ObsRO and PerfO.^43^^,^^58^ While generic PROs may be comparable across rare diseases, they may not be sensitive to specific aspects or changes in symptoms of individual rare diseases. Although disease-specific PROs may be more sensitive, their availability is limited for many rare diseases^66^ and they rarely provide the utility values required by HTA bodies.^44^^,^^68^

Furthermore, creating new COAs is time- and resource-intensive and establishing psychometric properties of newly developed measures is challenging in part due to the small patient populations.^44^ These challenges may lead to sparsity in the evidence necessary to select, modify, or develop and validate COAs as well as determine the utility of statistical analysis to guide decision-making which prevents regulators from conducting a comprehensive benefit-risk analysis of new therapies.^37^

### Recommendations

While these challenges exist, it is still important to capture patients' outcomes including their symptoms and function, in drug development. Improving patients’ quality of life may be the most important outcome in rare diseases with a chronic course and without cure.^61^^,^^69^

The rare disease context presents challenges as well as opportunities to develop and use cutting-edge innovations in COA for drug development. However, efforts to facilitate COA use for rare disease clinical trials should be balanced with the preservation of scientific integrity, regulatory requirements, operational feasibility, and financial implications.^42^ The following 11 recommendations, along with other existing PRO, trial design and regulatory guidance (see Table 2), are intended to facilitate the incorporation of COAs in drug development as well as generate high quality evidence to support decision making.^20^^,^^23^, ^24^, ^25^, ^26^^,^^42^^,^^56^^,^^70^, ^71^, ^72^, ^73^, ^74^, ^75^, ^76^, ^77^, ^78^, ^79^, ^80^, ^81^, ^82^

#### Recommendation 1: engage and involve key stakeholders early and regularly in the development and implementation of COA strategy

Early and continued engagement and involvement with stakeholders, including drug development teams, decision makers (regulators and HTA) and people with lived experience of the rare disease (patients, family members/caregivers, patient advocates) throughout the drug development process is important for the successful development of any new medicinal product.^37^^,^^75^^,^^83^^,^^84^ Successful drug development in rare disease requires ongoing partnership between stakeholders.^53^^,^^58^^,^^83^^,^^85^ Stakeholders can provide valuable input on the development and implementation of the COA strategy for clinical research. This is crucial because rare disease clinical trials often require novel approaches to trial design, and the evaluation of heterogenous patient-relevant outcomes and COAs to assess these outcomes which may be unfamiliar to decision-makers.^57^ It is vital that the goals of stakeholder partnerships, the drug development programme, and the COA are defined clearly and agreed from the outset by all stakeholders as individual groups may have different priorities and expectations.^53^

Patients, caregivers, clinicians, and patient organisations can provide valuable insights on lived experiences, priorities and preferences for treatment and the natural histories of rare diseases which may not be available in medical or academic literature.^57^^,^^58^^,^^86^ These insights may ensure that outcomes that are relevant and meaningful to patients are evaluated as endpoints in clinical trials–a first step in the development of a COA strategy. Patient organisations through their existing networks with patients and caregivers can facilitate the recruitment of patients for clinical trials and studies to understand patients’ lived experiences to help select concepts to evaluate as trial endpoints.^42^^,^^84^ Patients, caregivers, and individuals from patient organisations who contribute to clinical research do so while managing their personal health as well as financial challenges. They should be appropriately reimbursed for their time and out-of-pocket expenses related to their contribution to clinical research.^76^

Regulators and HTA organisations are keen to work with patients and patient organisations^87^^,^^88^ as well as provide scientific advice to sponsors and clinical trial teams.^20^ Regulators offer total or partial fee exemptions for scientific consultations for rare disease drug development.^18^^,^^21^ The EU-wide JCA facilitates the evaluation of new medicines and devices for sponsors by harmonising the process and producing a single report for all member states to support their national pricing and reimbursement decisions.^89^ In the UK, the MHRA and NICE have jointly developed an aligned pathway to streamline licencing and the value assessment processes so that decisions are published simultaneously, rather than consecutively, potentially accelerating patient access.^90^

#### Recommendation 2: ensure that the selected COA is fit for purpose

COA selection should start with the clarification of the concept(s) of interest and the context of use.^25^^,^^91^ The selected COA needs to be fit-for-purpose, i.e., appropriate with a clear rationale for intended use (study design and patient population), that reliably measures clinically relevant concepts which are important to patients, and generates data that can be communicated in a way that is accurate, interpretable, and not misleading.^25^^,^^91^

A conceptual framework should be developed to facilitate COA selection summarising (1) relevant experiences of patients and their representatives in the target population considering equity, diversity and inclusion (EDI), (2) specific concepts of interest targeted for assessment, (3) type(s) of COA proposed for each concept of interest, and (4) a representation of how the particular COA is intended to work in order to generate a score reflecting the concept of interest.^25^ Sponsors should ensure that stakeholders including patients, caregivers/family members, clinicians, and regulators are involved in the development of the conceptual framework, mapping of concepts to COA tools and the selection process.^25^^,^^92^^,^^93^

Sponsors should also demonstrate that consideration has been given to the appropriateness of COA concepts, measures, endpoints, and data, even when these COAs are not intended to support labelling claims. For example, early conduct of qualitative studies (concept elicitation and cognitive debriefing) with patient representatives from the target population for the drug can be used to select the concepts of interest and establish the content validity of selected measures.^37^^,^^58^ Sponsors should prioritise endpoints and outcomes that are proximal to the condition and most likely to demonstrate treatment effect to maximise the chances of regulatory success. More distal outcomes can be collected as these may be relevant to non-regulatory stakeholders (e.g., payers, healthcare providers, and patients).

In general, PROs are the most used COA in clinical trials as they provide insights directly from trial participants.^94^ However, in paediatric settings and conditions associated with debilitating and/or progressive cognitive, speech, or motor dysfunction, traditional self-report methods may not be useful, and the use of specialised reporting strategies or other types of COA tools such as ObsROs, PerfOs, and/or ClinROs are likely to be required.^61^^,^^72^ Therefore, the decision on COA strategy should consider the context of use as well as the concepts of interest.

#### Recommendation 3: check COA databases for relevant existing COAs and consider using item libraries particularly in the absence of predefined measure(s)

Relevant databases such as the Critical Path Institute's Rare Disease COA Resource^95^^,^^96^ and Mapi Research Trust's ePROVIDE database can be searched for relevant COAs.^92^ In the absence of predefined disease-specific COAs that are fit-for-purpose i.e., appropriate for the context of use and concepts of interest, consider selecting appropriate items from validated item libraries liaising with regulators to ensure that this approach is acceptable.^61^^,^^97^ PRO item libraries include the European Organisation for Research and Treatment of Cancer (EORTC) Item Library,^98^ Symptom Burden Questionnaire (SBQ),^99^ the Patient-Reported Outcomes Measurement Information System (PROMIS),^100^ the Functional Assessment of Chronic Illness Therapy (FACIT) Item Library,^101^ and the National Cancer Institute's Patient-Reported Outcomes version of the Common Terminology Criteria for Adverse Events (PRO-CTCAE®).^102^ The use of item libraries facilitates the selection of items most relevant to the context of use, thereby minimising respondent burden.^43^^,^^61^^,^^75^ Computer adaptive testing (CAT) can be used with item banks to further tailor PRO assessments for individual respondents.^75^^,^^77^ Domains such as fatigue, physical function, pain interference, mobility, sleep disturbance, and cognitive function have been assessed in some rare disease studies using item banks and CATs.^103^ The use of CAT is recommended particularly when the health status of the patient population is very poor (to reduce burden), which is an important consideration for rare disease clinical trials.^104^ An added advantage is that the use of CAT can provide PRO assessments that are less affected by floor and ceiling effects compared to fixed-length measures.^104^

When item lists are derived from item libraries that contain validated questionnaires, it may not be necessary to conduct additional psychometric testing.^97^ However, pilot testing of the selected items is recommended, to ensure relevance and comprehensibility for the target population. If the item list is intended to be administered in a new population, further comparative validation testing may be required prior to their use in trials to ensure that the psychometric properties are retained.^97^ Items targeting specific outcomes or symptoms of rare diseases may not be available in these item libraries. This may require the development of additional items which would need psychometric validation. New items should be generated in collaboration with the instrument developers to facilitate the use and extension of existing item libraries.

#### Recommendation 4: consider adapting existing measures before embarking on developing new ones

Another approach to outcome measurement in rare diseases is to adapt existing measures from a phenotypically similar disease to the rare disease context. For example, the Unified Parkinson's Disease Rating Scale (UPDRS)-2, originally developed for and widely used in Parkinson's disease (PD), was adapted for use in pantothenate kinase-associated neurodegeneration (PKAN), a rare disease that causes similar motor and speech deficits although with a completely different aetiology.^61^ Relevant guidance on the modification and development of COA should be consulted.^25^^,^^105^^,^^106^

It is recommended that psychometric and other evaluations to assess a COA's fitness for purpose should be conducted prior to use in a registration trial's endpoint. If it is not feasible to validate the COA in earlier trials, a stand-alone observational study may be conducted before the registration trial(s).^25^ Given the small population sizes and the limited available data in the rare disease context, pragmatism and innovation are needed in rare disease research, while maintaining high methodological standards.^58^ A mixed-methods psychometric approach which incorporates qualitative and quantitative data from multiple sources including interviews, surveys, social media, natural disease history, and patient advocacy group discussion forums has been recommended.^58^^,^^61^^,^^107^, ^108^, ^109^ The content validity of any COA needs to be determined as a priority.^25^^,^^58^^,^^110^ It might be necessary to update existing evidence of content validity when new advances in treatment are significant enough to change the patient experience of their condition and the concepts of interest.^61^

#### Recommendation 5: where appropriate, consider measuring core sets of outcomes and/or developing a COA that is suitable for use across related rare diseases

It is not feasible to develop condition-specific COA tools for every rare disease (see Recommendations 3 and Recommendations 4). However, groups of rare diseases often share similarities in terms of symptoms and disease progression and cross-cutting concepts that are meaningful and relevant across multiple rare diseases may give rise to COAs that are fit for purpose among a set (or more than one set) of rare diseases. Outcomes such as physical function, disease progression, and school attendance/work participation may be relevant across several rare diseases.

Where appropriate, consider using/developing concept-specific measures, which may be applicable across a ‘family or cluster of rare diseases’ (e.g., autoimmune diseases that share chronic fatigue or pruritus as a chief complaint, or neuromuscular diseases associated with loss of ambulation).^58^^,^^111^^,^^112^ For instance, the Overall Disability Scale (R-ODS) was developed to assess activity and social participation limitations in patients with Guillain-Barré syndrome (GBS), chronic inflammatory demyelinating polyradiculoneuropathy (CIDP), and gammopathy-related polyneuropathy (MGUSP).^112^ This approach might be particularly suitable for novel trial designs such as umbrella and basket trials.^60^^,^^113^ The use of a ‘core’ set of outcomes can facilitate the pooling and comparing of COA data across different genetic or other subgroups where appropriate.^114^^,^^115^ However, this is not a one-size-fits-all approach. Sponsors need to critically evaluate the relevance and suitability of any set of outcomes to their specific clinical programme.

#### Recommendation 6: determine early the approach to data analysis particularly when usings COAs as efficacy endpoints

Early agreement on approach to data analysis such as power calculation, estimands, and handling of missing data is essential. The FDA has recently published draft guidance to facilitate the use of Bayesian methodologies in clinical trials of drugs and biologics, helping drug developers make better use of available data and conduct more efficient clinical trials.^116^ Bayesian methodologies are particularly relevant to the rare disease context where the patient populations are typically small as they facilitate the incorporation of pre-study data from sources such as natural history studies (see Recommendation 10). Descriptive endpoint analyses have been used in scenarios with very limited sample sizes such as ultra-rare diseases. In such cases, additional supporting data on treatment benefit should be collected (see Recommendation 9).

The assessment of meaningful change at subject and group levels is important, especially when using COAs as efficacy endpoints in clinical trials. For COAs to be maximally useful to regulators, payers, and patients, it is important to define what constitutes a meaningful change, both qualitatively and quantitatively.^26^ Thresholds for each COA should be predefined, justified, and where possible, those involved in the data collection (clinicians, caregivers, research staff) should not be aware of the threshold definitions to minimise bias.^26^ A clear distinction should be made between within-patient change (individual benefit) and between-group differences (treatment efficacy).^117^ Anchor-based approaches which link changes in COA score to an external ‘anchor’ such as patient-reported global impression of change scales with known clinical interpretations are generally preferred. Well-designed global impression of change scales that align with the target concept of interest should be used. Sponsors should consider triangulation of approaches (anchor-based, distribution-based, and qualitative) to enhance confidence in the estimated threshold. Where anchor-based methods for assessing meaningful change in COA scores are not feasible (due to limited sample size), robust in-trial interviews should be conducted (see Recommendation 9).

Proactively educating trial participants and study site personnel prior to data collection on the purpose of the trial COA may foster deeper engagement and reduce the likelihood of missing data.^75^ Sometimes conceptual overlaps across measures cannot be avoided entirely and may be helpful for validation purposes. Participant education provides the opportunity to explain the rationale and emphasise the importance of completing all assessments, even if some redundancy exists.

#### Recommendation 7: consider the methodological approach and data requirements for economic evaluations early

Rare disease therapies may be conditionally approved while clinical trial follow-up or real-world data collection is ongoing.^118^ The generation and fluid integration of real-world evidence to dynamic economic models reflecting the latest available evidence can improve the evaluation of these therapies.^118^^,^^119^

The impacts of disease and treatment on a patient's family members and informal caregivers (“family spillover effects”) and the importance of incorporating these impacts in HTAs and cost-effectiveness analyses (CEAs) of therapies are acknowledged and well described in literature.^120^^,^^121^ However, despite the existence of tools and guidance^122^^,^^123^ to facilitate the inclusion of spillover effects in HTAs, this is typically not done as it is commonly perceived as challenging.^118^^,^^123^, ^124^, ^125^, ^126^ Input from patients, family members/caregivers, HTA bodies, and health economists should be sought early during trial design and regularly during trial delivery as required (see Recommendation 1). Further research in collaboration with HTA bodies and patient communities is required to develop the evidence base to support the integration of broader impacts of rare disease therapies in CEAs.^118^^,^^123^^,^^127^, ^128^, ^129^

#### Recommendation 8: consider using digital health technologies to enhance data collection and analysis

The use of digital health technologies (DHTs) can facilitate the decentralisation of clinical trials, providing sponsors and clinical trial teams the opportunity to collect multiple COA-related longitudinal data and identify adverse events remotely.^42^^,^^130^^,^^131^ Decentralised, patient-friendly trial designs with robust participant support systems are essential for success. Data from DHTs can be combined with COA data, and other clinical trial data.

Most wearable devices require minimal input from participants and can measure parameters such as heart rate, sleep, physical activity, electrocardiography, and oxygen saturation.^132^ For example, the device agnostic Stride velocity 95th centile (SV95c) has received qualification by the EMA for use as a primary endpoint for Duchenne muscular dystrophy (DMD) to measure ambulatory function.^133^ Nevertheless, some individuals may find these devices intrusive and there may be issues with their accuracy, algorithms, and calibration and concerns around data privacy and security.^42^ Therefore, the selection, verification, validation, and usability evaluations of DHTs need to be robust.^134^^,^^135^ Sponsors should check early with regulators whether DHTs require regulatory endorsement and qualification before being used for clinical research.^136^

The use of electronic COA (eCOA) may provide several potential benefits such as reducing respondent burden, reducing human errors, and facilitating the integration with other forms of data. Video-based COAs may better capture the impact of rare diseases and facilitate the assessment of specific parameters or outcomes in certain settings. For instance, in diseases where mobility is impacted, such as gait and stride length, video-based COA can add a powerful dimension to COA in rare disease drug development.^57^^,^^137^ Video-based COA could be implemented *de novo* in clinical trials to demonstrate treatment benefit while historical videos of disease progression (including those filmed by non-experts) could serve as external comparators. While there are challenges with video-based COA, technological advances can automate the analysis of video recordings and reduce human assessment bias.^137^^,^^138^ However, there is a need to consider potential issues relating to access to technology when using eCOAs and video-based COAs.^42^^,^^139^

Where appropriate, the capabilities of artificial intelligence (AI) could be leveraged for COAs in rare disease drug development.^71^^,^^79^^,^^132^^,^^140^ AI should be employed to complement and not replace the use of existing tools and methods. However, there are important issues with AI including scientific rigour, reliability, accuracy, representativeness and equity issues, ethics, and latent capabilities that raise safety and biosecurity concerns and create regulatory challenges.^71^^,^^132^^,^^140^, ^141^, ^142^, ^143^ Therefore, robust strategies are required to mitigate these risks.^79^^,^^141^ The regulators have published relevant guidance on the use of novel methodologies, AI in drug development.^81^^,^^144^, ^145^, ^146^

#### Recommendation 9: consider capturing other forms of patient experience data in addition to COAs in rare disease drug development

Data from COA-based endpoints might not capture all the meaningful aspects of health or demonstrate the impact of a treatment in the rare disease context. Therefore, consider collecting other forms of patient experience data in addition to COA for rare disease clinical trials.^58^ Patient diaries providing a record of the patient/caregiver perspective of their day-to-day experiences and thoughts relating to their health and medical condition can be used.^147^ Qualitative research methods can provide valuable patient experience data, and patients enrolled in clinical trials and their caregivers may be useful sources for qualitative data collection.^29^^,^^58^^,^^148^ Regulators and HTA bodies are increasingly considering and using in-trial interview data, particularly for rare diseases, in their evaluation of new therapies.^149^ In-trial interviews (entry and exit) can offer rich opportunities to gain deeper insights into the disease, treatment experience, meaningful changes in symptoms or treatment benefits, and to support the interpretation of meaningful score changes.^149^^,^^150^ Entry interviews could provide a baseline for patient and caregiver lived experiences before a trial starts. Consecutive “embedded” interviews conducted during a clinical trial, in addition to exit interviews could capture the evolution of patients’ treatment experience and perception of treatment benefit throughout the trial.^58^^,^^151^^,^^152^ Ideally, some interviews should coincide with the administration of COA tools as this could provide a valuable opportunity to explore COA data further and obtain rich contextual information. However, care should be taken to minimise the risk of these embedded interviews impacting the integrity of the trial/primary endpoint.^24^^,^^148^ Interviews should be conducted by trained qualitative experts and not site staff.^148^ Potential patient burden needs to be considered when deciding the frequency of these interviews as this could affect the quality of the data obtained.^24^ Early planning and alignment of in-trial interview objectives with regulatory and HTA requirements are needed to enhance the relevance and impact of qualitative evidence in drug development.^149^

Individualised assessments and tools such as Goal Attainment Scaling (GAS) may be used to capture changes that matter to patients with rare diseases particularly ultra-rare diseases when validated disease-specific measures are unavailable or impractical.^153^^,^^154^ GAS is an individualised outcome measure in which each patient (and or caregiver) defines a small set of priority goals in collaboration with a clinician. It provides both qualitative and quantitative information on progress towards goal attainment after an intervention or treatment and allows assessment of clinically meaningful change that is unique to each patient.^153^, ^154^, ^155^ In these ultra-rare contexts, demonstrating efficacy means showing that a patient has meaningfully improved or stabilised relative to their pre-treatment trajectory, rather than relying solely on traditional group comparisons or natural history studies. However, there is a need for standardisation in the implementation and reporting of individualised assessments to enhance reproducibility and assessment of its validity which would be important for regulatory and HTA needs.^154^^,^^156^

Natural history studies may be conducted before or in parallel with clinical trials and provide additional information to support new drug applications (NDAs) as well as generate post-marketing data. While randomised controlled trials remain the gold standard for drug development, in the rare disease context, alternative treatments to use as comparators are typically unavailable, and withholding potentially life-saving treatment from a control group or the use of placebo would be unethical. As such, natural history studies have the potential to be used as external comparators for rare disease clinical trials.^49^^,^^157^ For instance, Brineura for the treatment of late infantile neuronal ceroid lipofuscinosis type 2 (CLN2) was approved by the FDA and EMA based on a single-arm pivotal trial which utilised an external control of untreated patients from a natural history study.^49^ Sponsors and trial teams should therefore ensure that the COA tools they select, adapt, or develop are capable of capturing data that could be directly compared to natural history data if this would serve as an external comparator.^158^ Where regulatory guidance on the use of natural history studies exist, these should be followed.^159^^,^^160^

#### Recommendation 10: provide trial updates and share trial findings including COA data with study participants and rare disease communities to maintain research partnerships

Trial participants, patients, and caregivers generally value communication regarding progress of ongoing trials as well as other relevant research activities.^161^, ^162^, ^163^ Sponsors and clinical trial teams should co-develop a communications plan early with patient partners, caregivers, and representatives of patient organisations. They should provide trial updates and disseminate trial findings (including COA data) to trial participants and the public to close the feedback loop using appropriate language and channels including patient organisations and online forums.^42^^,^^164^, ^165^, ^166^ The timing and content of such dissemination should be considered carefully so as not to compromise the integrity of the trial data due to functional unblinding and bias. Post-trial communication can help maintain relationships with rare disease communities and facilitate future research including the generation of real-world evidence for new therapies (see Recommendation 1).^167^, ^168^, ^169^

#### Recommendation 11: report experiences of development and implementation of COA

Sponsors and trial delivery teams should report their experiences of developing and implementing their COA strategies for rare disease clinical trials. Such publications can provide valuable insights to guide other teams in their future work as well as generate stronger evidence to support the use of novel strategies, some of which have been described in this article.^58^ The creation of a shared data repository made available to collaborators could unlock collective insight, accelerate discovery, and transform isolated datasets into a harmonised unit for rare disease breakthroughs.

## Discussion

Decisions about the appropriate approaches to employ for clinical outcome assessment is particularly challenging for rare disease drug development. Empirical evidence, as well as current information on the expectations and perspectives of regulators, patients, payers, industry and clinical trialists is not readily available. This study aims to address this information gap by reviewing published literature and convening a multistakeholder workshop with stakeholders who possess suitable experience and expertise to increase the relevance, and promote the uptake of the final recommendations.

Drawing on the most current evidence in literature and the stakeholder insights, we provide a structured set of 11 practical recommendations to facilitate COA utilisation and optimisation in rare disease drug development. We highlight various issues and provide suggestions for sponsors, trial delivery teams, and researchers to consider when designing clinical trials in the rare disease context.

While PROs are the most used COA, most of the recommendations provided here apply to and should be considered for other types of COAs. Decisions about COA strategy and trial design should be made with input from all stakeholders including patients, caregivers, patient organisations, researchers including experts in biostatistics and health economics, industry, regulators, and HTA agencies. Proactive, early, and regular interactions with stakeholders is essential to minimise the risk that trial outcomes may not meet the necessary requirements for regulatory approvals, reimbursement, and patient centred care.^37^

Beyond the need to optimise COA utilisation in rare disease trials, there are broader issues that need to be addressed such as equitable access to therapies (particularly for paediatric patients), market failure due to several factors including high manufacturing costs and regulatory challenges, and ethical challenges with the use of novel technologies.^170^ Again, stakeholders need to work collaboratively to successfully address these issues.

This work has strengths and limitations. Key strengths include the conduct of a targeted literature review that captured the current evidence on COA in rare disease drug development. Targeted literature review methods have been used to support similar published work.^171^ The PubMed search was sufficiently sensitive as it identified a key paper which was subsequently used for our pearl search on Google Scholar. The Google Scholar search strategy yielded several recent and relevant articles in line with published evidence on the effectiveness of pearl growing methodology.^38^, ^39^, ^40^ The multistakeholder workshop had appropriate stakeholder representation involving several patients and caregivers with lived experiences and professional experts with substantial experience in the field whose perspectives and suggestions strengthened the recommendations provided. The anonymity of the final vote addressed the risk of group conformity.^172^ The provision of examples illustrating the use of COAs in rare disease trials provide context and aid understanding of the issues and recommendations.

The limitations of the review are typical for targeted literature reviews, and these included the restriction of the database search to PubMed and the use of simple Boolean searches which might not have identified all the relevant literature. The use of a single reviewer screening also carried a higher risk of selection bias compared to systematic reviews.

Another limitation was having workshop participants exclusively from the UK and the US. We invited some experts from other countries, who either declined due to prior commitments or were unable to attend after initially accepting due to unforeseen personal reasons. While our goal was to develop recommendations applicable to most geographical contexts, we recognise that sponsors might face challenges implementing Recommendation 8 (pertaining to the use of digital technologies) in low- and middle-income countries and high-income countries with study participants from low socioeconomic backgrounds. Representation from other countries might have provided additional insights. We have provided references to resources that explore the issue of access in greater detail and provide recommendations for sponsors and research teams to consider.^42^^,^^139^

The recommendations provided here should be used in conjunction with other available guidance to facilitate COA utilisation in rare disease clinical trials.^12^^,^^20^^,^^42^^,^^56^^,^^70^, ^71^, ^72^, ^73^, ^74^, ^75^, ^76^, ^77^, ^78^, ^79^, ^80^^,^^83^^,^^141^ Trial sponsors, trial delivery teams, and researchers should consult broader regulatory guidance. Early engagement with regulatory agencies is recommended.^23^, ^24^, ^25^, ^26^^,^^81^

## Contributors

Concept and design: OLA, NKM, JDP, MJC, TGB, (Study Group author: DJ). Recruitment of participants: OLA, TGB, NKM, AJM, CC, KL and Study Group Authors: LAW, SS, HC, SJB, DJ. Workshop participation: OLA, NKM, TGB, MJC, SA, FA, TB, LB, RC, CC, BMC, MD, MDD, JE, LF, SG, CG, AH, OAI, KL, KLB, SPL, CM, MM, AJM, FM, RM, LTM, DJO, HO, LO, KP, KRR, VS, (Study Group author: DJ). Data acquisition, analysis, interpretation, generation of initial recommendations: OLA, MJC, BMC, HO, and DJO. Drafting of the manuscript: OLA. Input in development of final recommendations: All authors. Survey development and analysis: SCR and OLA. Critical revision of the manuscript for intellectual content: All main authors (OLA, JDP, SCR, RML, NKM, TGB, MJC, SA, FA, TB, LB, RC, CC, BMC, MD, MDD, JE, LF, SG, CG, AH, OAI, KL, KLB, SPL, CM, MM, AJM, FM, RM, LTM, DJO, HO, LO, KP, KRR, VS). The Study Group authors (DJ, MC, SG, LAW, ARH, SS, HC, SJB, VH, BH, MH, MRH, AR, CT) also reviewed the manuscript for intellectual content.

All authors read and approved the final version for publication. OLA secured ethical approval for the workshop. OLA and LAW accessed and verified the underlying data. OLA takes responsibility for the integrity of the data and the accuracy of the data analysis. Study Group refers to the collaborator group indicated as LifeArc Accelerating Rare Disease Trials (ARDT) centre within the authorship line.

## Data sharing statement

Aggregated voting data supporting the inclusion of the final recommendations are included in the article and its Supplementary File. No individual level data are publicly available in accordance with the ethical approval granted for the study.

## Declaration of interests

OLA receives funding from the National Institute for Health and Care Research (NIHR) Birmingham Biomedical Research Centre (BRC), NIHR Blood and Transplant Research Unit (BTRU) in Precision Transplant and Cellular Therapeutics, NIHR Applied Research Collaboration (ARC) West Midlands at the University of Birmingham and University Hospitals Birmingham NHS Foundation, Innovate UK (part of UK Research and Innovation), LifeArc, Health Foundation, Merck, Gilead Sciences Ltd, Anthony Nolan, Sarcoma UK, and GSK. He declares personal fees from Gilead Sciences Ltd, Merck, GlaxoSmithKline, and Boehringer Ingelheim outside the submitted work. JDP declares funding and support from US FDA, UKRI, Orphalan, Gemeinsamer Bundesausschuss, GIMEMA, European Network of Reference Centres. He also declares consultancy fees for Orphalan, DayOne Pharmaceuticals, Ionis Pharmaceuticals, Beta6, and Northwestern University. MJC is Director of the Centre for Patient Reported Outcomes Research, Deputy Director of the Birmingham Health Partners Centre for Regulatory Science and Innovation and is a National Institute for Health and Care Research (NIHR) Senior Investigator and Fellow of the Academy of Medical Sciences. MJC receives funding from the National Institute for Health and Care Research (NIHR), UK Research and Innovation (UKRI), NIHR Birmingham Biomedical Research Centre (BRC), NIHR ARC West Midlands, European Regional Development Fund, Innovate UK (part of UKRI), Merck, GSK and Gilead. MC has received personal fees from Aparito Ltd, Boehringer Ingelheim, CIS Oncology, EuroQoL, Gilead, Halfloop, ICON, Merck, Pfizer, Shionogi B.V. and Vertex outside the submitted work. TB is an employee of Alexion, AstraZeneca Rare Disease, and holds stock or stock options in AstraZeneca. KL is an employee of Alexion, AstraZeneca, and holds stock or stock options in AstraZeneca. AJM is funded by LifeArc and Health and Social Care Research and Development (HSC R&D) division (COM/5802/24), Northern Ireland. AJM is also Director of the Northern Ireland Rare Disease Partnership. SPL is an employee of UCB and holds stock/stock options in UCB, Pfizer and BMS. HO is an employee of BioMarin Pharmaceutical Inc and holds stocks in Arcutis Biotherapeutics, BioMarin Pharmaceutical Inc, Eli Lilly, and Tenaya Therapeutics. DJO is an employee of the Association of the British Pharmaceutical Industry (ABPI). BMC is an employee of GlaxoSmithKline. RML has received sponsorship from Roche and Biogen, and has done consultancy work for Novartis, Biogen, Roche and the Health Learning and Research Consulting Group. MM is an employee of Takeda Pharmaceuticals and holds stock in Takeda Pharmaceuticals. She declares licencing fees from the development of the Observer-Reported Communication Ability (ORCA) measure. RM receives funding from the Manchester Biomedical Research Centre and the Medical Research Council, United Kingdom. TGB is an NIHR Senior Investigator and has had consultancy appointments with Novo Nordisk and Amylyx Pharmaceuticals. He declares funding from LifeArc. He has received lecture honoraria from Sanofi Aventis and Alexion Healthcare. SJB receives funding from the National Institute for Health and Care Research, the NIHR Birmingham Biomedical Research Centre (BRC), NIHR Research Support Service, Innovate UK (part of UK Research and Innovation), Gilead and LifeArc. MRH receives funding from LifeArc. VH declares consulting fees from FIPRA—Together4RD project. CT declares grant funding from Duchenne UK. SG receives funding from LifeArc and UKRI. LO declares consultancy fees from Intent Health, Vertex pharmaceuticals, Astellas Pharma, Boehringer Ingelheim, and Biohaven pharmaceuticals. She had educational grant from CSL vifor, sits on the advisory board of Syneos Health and is an expert reviewer for Harrington Discovery institute. BH receives funding from LifeArc. All remaining co-authors declare no competing interests.

## Appendix A Supplementary data

Supplementary Material

Collab Authors
