# Supplementary material for: Recommendations for the use of clinical outcome assessments in rare disease drug development
Source: eClinicalMedicine. 2026 Jul 20;98:104073. doi: 10.1016/j.eclinm.2026.104073 (PMC13393705; doi:10.1016/j.eclinm.2026.104073)
Supplement: Supplementary Material [file mmc1.docx]

**Supplementary file**

**Targeted literature review**

**Inclusion and exclusion criteria**

*Inclusion criteria*

- Qualitative and quantitative studies focused on the use of any type of COA for all stages and phases of rare disease drug development.
- Studies that report the use of COA for regulatory, product labelling, and reimbursement decisions specifically in the rare disease context.
- Studies involving adult and paediatric patients with any rare diseases and their family caregivers
- Regulatory and health technology assessment guidance relevant to COA implementation for rare disease drug development, regulatory approval and reimbursement.

*Exclusion criteria*

- Studies solely reporting the development or implementation of COA measures
- Articles focused on clinical aspects of rare disease clinical research with no COA component
- Abstracts and study protocols without PRO data.

| **Table 1. PubMed Search strategy and entries retrieved** | | | |
| --- | --- | --- | --- |
|  | **Search terms for literature review** | **Initial search**  **26/08/2025** | **Update search 27/02/2026** |
| 1 | rare disease [Title/Abstract]) AND (patient reported outcome [Title/Abstract]) | 76 entries | 14 additional entries |
| 2 | rare disease [Title/Abstract]) AND (clinician reported outcome [Title/Abstract] OR (ClinRO [Title/Abstract]) | 41 entries | 8 additional entries |
| 3 | rare disease [Title/Abstract]) AND (observer reported outcome [Title/Abstract] OR (ObsRO [Title/Abstract]) | 24 entries | 2 additional entries |
| 4 | rare disease [Title/Abstract]) AND (performance outcome [Title/Abstract] OR (PerfO [Title/Abstract]) | 20 entries | 5 additional entries |

**Selection process, data analysis and synthesis**

Search records were exported to EndNote. OLA screened all the titles and abstracts following the predetermined eligibility criteria. Full-text articles were retrieved for studies potentially eligible for inclusion and OLA evaluated all the publications.

No formal quality assessment of included publication was conducted as this was not a systematic review.

The final set of publications were grouped by OLA based on publication type i.e. clinical trials, qualitative studies, health economic evaluation studies, regulatory/HTA guidance, and other quantitative studies.

Finally, relevant data on (i) COA challenges and benefits in rare disease drug development (ii) COA trial/study results (iii) regulatory guidance/recommendations and (iv) rare disease statistics were extracted and narratively synthesised by OLA.

**Summary of workshop discussions**

The workshop discussions were organised as three sessions all on the same day, involving all the workshop participants. The key issues and considerations identified in the literature review were discussed during the relevant session. The sessions were focused on different aspects of COA assessment. Therefore, there was no active attempt to carry over the discussions from one session to another except in situations when certain points relevant for a later session were brought up by a workshop participant in a previous session and vice versa.

*Session 1: What outcomes to measure and how this should be done.*

During this session, participants discussed the difficulties identifying appropriate measures to use for rare disease trials due to the sheer number of conditions often with subtypes. The practicalities/feasibility of developing bespoke measures for each disease was discussed. Potential solutions such as checking databases for existing measures that are suitable, the use of item libraries, and development of new measures were also discussed. These map to Recommendations 2-5.

*Session 2: Important considerations for COA for rare disease drug development*

Participants focused on a range of considerations for COA rare disease drug development. They discussed the need for greater engagement, collaborative working and information sharing with stakeholders including patients and caregivers. Patient advocates highlighted to need to recognise and appropriately reimburse their support for clinical research. Participants also discussed the need to consider early how the data collected would support economic evaluations/HTA assessments of therapies particularly as accelerated approvals may be granted while awaiting further clinical data. The need to account for spillover effects which is particularly important for rare diseases was also discussed. The limits of COA were acknowledged, and the collection of other patient experience data was suggested. The discussions during this session map to final Recommendations 1, 6, 7, 9-11.

*Session 3: Future developments in outcome measurement in drug development*

This was a horizon scanning session where participants discussed the future developments for COA. The discussion was mainly about the potential use of technology innovations such as artificial intelligence and wearable devices to enhance drug development. The discussions during this session map to Recommendation 8.

**Smart Survey data**

The survey was completed by 28 participants. Of the 28, 10 were patients/patient advocates/caregivers, 8 were researchers/clinical trialists, 6 were industry experts and 3 were regulatory and HTA experts. One participant self-identified as COA expert.

All the 12 recommendations (10 initial and two additional recommendations suggested by co-authors) achieved the *a prior* threshold of at least 70% votes for inclusion (see screenshots below). The additional recommendation relating to natural history studies received the lowest vote at 82%. This recommendation was originally a component of the present Recommendation 9 (formerly Recommendation 8) focused on the collection of patient experience data (PED) but was proposed as a standalone recommendation based on earlier co-author feedback. Several co-authors noted that although they agree with its message, they would prefer it was part of Recommendation 9. Based on this feedback it was re-incorporated with Recommendation 9.


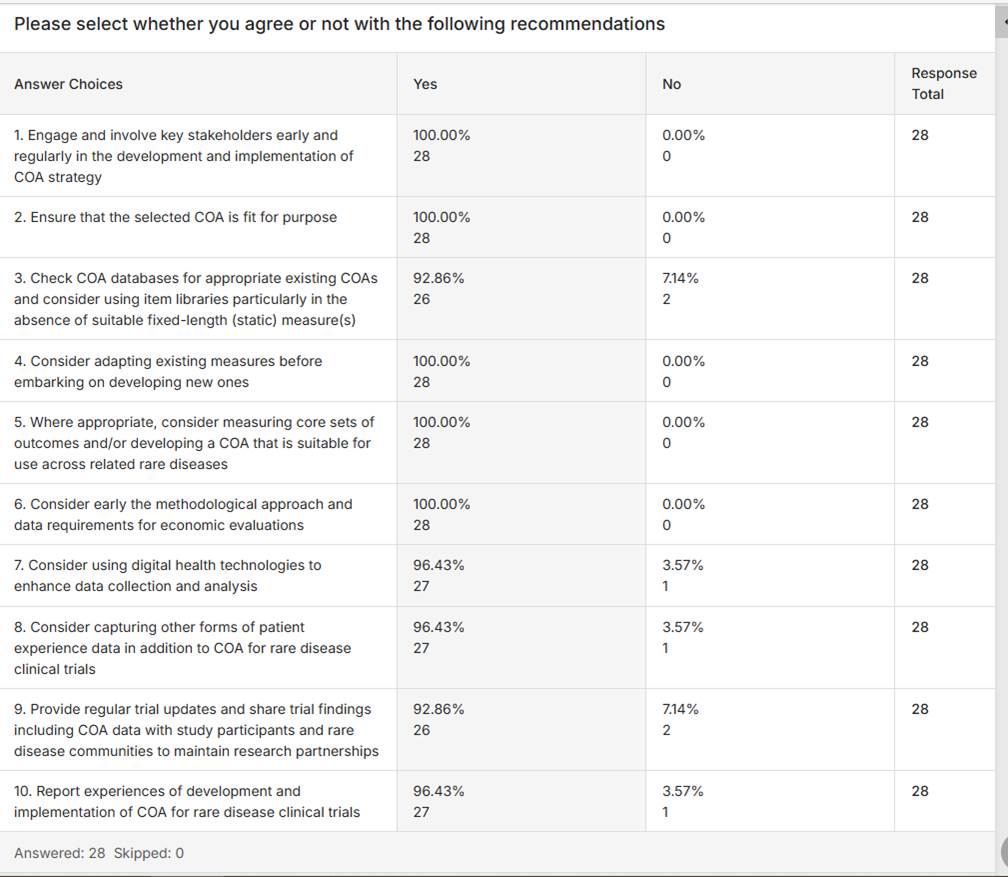


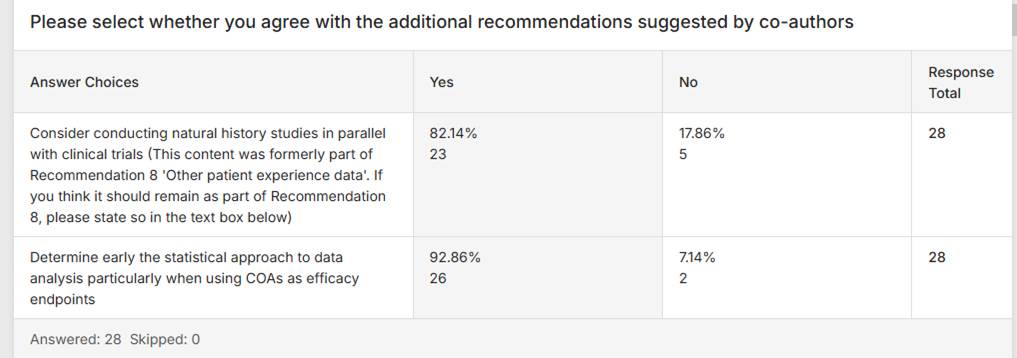


**LifeArc Accelerating Rare Disease Trials [ARDT] centre Group Author List**

| **First name** | **Middle name or initial** | **Surname** |
| --- | --- | --- |
| David |  | Jones |
| Michael |  | Clarke |
| Hayley |  | Comins |
| Simon |  | Gates |
| Amber | R | Hart |
| Victoria |  | Hedley |
| Blánaid |  | Hicks |
| Martin | R | Higgs |
| Matthew |  | Hosken |
| Ameeta |  | Retzer |
| Sarah |  | Scullion |
| Laura | A | Wyatt |
| Steven | J | Blackburn |
| Catherine |  | Turner |
