# Supplementary material for: Recommendations for the use of clinical outcome assessments in rare disease drug development
Source: eClinicalMedicine. 2026 Jul 20;98:104073. doi: 10.1016/j.eclinm.2026.104073 (PMC13393705; doi:10.1016/j.eclinm.2026.104073)
Supplement: Collab Authors [file mmc2.docx]

**PubMed LifeArc Accelerating Rare Disease Trials [ARDT] centre Group Author List**

| **First names** | **Surname** |
| --- | --- |
| David | Jones |
| Michael | Clarke |
| Hayley | Comins |
| Simon | Gates |
| Amber R | Hart |
| Victoria | Hedley |
| Blánaid | Hicks |
| Martin R | Higgs |
| Matthew | Hosken |
| Ameeta | Retzer |
| Sarah | Scullion |
| Laura A | Wyatt |
| Steven J | Blackburn |
| Catherine | Turner |
